# High-volume workflow and performance comparisons for *Chlamydia trachomatis* and *Neisseria gonorrhoeae* testing using automated molecular platforms

**DOI:** 10.1186/s12879-019-4442-0

**Published:** 2019-09-11

**Authors:** André Frontzek, Gudrun Aretzweiler, Daniela Winkens, Dana Duncan, Elizabeth M. Marlowe

**Affiliations:** 1 Molecular Biology (PCR) Department, Limbach Group, Labor Mönchengladbach, MVZ Dr. Stein + Kollegen GbR, Tomphecke 45, 41169 Mönchengladbach, North Rhine-Westphalia Germany; 2 Medical and Scientific Affairs, Roche Molecular Systems, Inc., Pleasanton, California, USA

**Keywords:** *Chlamydia trachomatis*, *Neisseria gonorrhoeae*, Sexually transmitted diseases, Automation, Laboratory, Sensitivity and specificity, Workflow, Nucleic acid amplification techniques, Body fluids

## Abstract

**Background:**

The global burden of sexually transmitted infections (STIs) is high and there have been reports of increasing chlamydial and gonorrheal infections. High-volume screening programs for *Chlamydia trachomatis* (CT) and *Neisseria gonorrhoeae* (NG) are an important component of STI control. This study evaluated the high-volume workflow and performance of the **cobas®** CT/NG assay for use on the automated Roche **cobas®** 6800 system, with the **cobas p** 480 instrument for pre-analytics, compared with the Aptima Combo 2 assay on the Hologic Panther system.

**Methods:**

High-volume workflow and performance were evaluated using paired female urine specimens. Workflow analysis (*n* = 376) included hands-on time (HoT), number of manual interventions, and time to first and last results. For performance assessment, paired results from the **cobas** CT/NG and Aptima Combo 2 assays, for both CT and NG, were compared and two-sided 95% confidence intervals calculated to provide estimates of positive percent agreement (PPA), negative percent agreement (NPA), and overall percent agreement (OPA) between the tests. McNemar’s test was used for significance testing.

**Results:**

Pre-analytical preparations and system start-up on the **cobas** 6800 system required 00:27:38 (hr:min:sec) HoT whilst the Panther system required 00:30:43. The **cobas** 6800 system required eight interactions and 00:43:59 HoT to process 376 samples. The Panther system required six interactions and 00:39:10 HoT. Time to first results was 02:53:00 on the **cobas** c6800 system for 96 samples and 03:28:29 on the Panther system for five samples. The **cobas** 6800 system delivered all 376 results 3 h faster than the Panther system (07:45:26 and 10:47:30, respectively). The performance correlation between both assays was high (PPA, NPA and OPA > 99% for both CT and NG). McNemar’s test revealed no statistically significant difference between the assays.

**Conclusion:**

For high-volume automated CT/NG testing, both the **cobas** 6800 system and Panther system provided accurate results. Although less manual intervention steps were needed for the Panther system, improved turnaround time was obtained with the **cobas** 6800 system with less risk for contamination. The additional testing capacity on the **cobas** 6800 system would allow a growing service to deliver more results in a single shift.

**Supplementary information:**

**Supplementary information** accompanies this paper at (10.1186/s12879-019-4442-0).

## Background

The global burden of sexually transmitted infections (STIs) is high [1]. Recently, data from the USA have identified year-over-year increases in chlamydial and gonorrheal infections [2]. Factors contributing to this include: the targeted expansion of screening practices; wider acceptance of HIV pre-exposure prophylaxis (with the resultant increase in condomless sex); and social networks with increased connectivity and the potential for sexual partners [2–4]. In this challenging environment, high-volume screening programs for *Chlamydia trachomatis* (CT) and *Neisseria gonorrhoeae* (NG) are an important component of STI control [2, 5–8].

Nucleic acid amplification tests are highly sensitive and the preferred tests for STI screening and diagnosis [5, 6, 8]. The **cobas®** CT/NG and Aptima Combo 2 assays are commercially available nucleic acid amplification tests with utility across different specimen types, including female urine [9, 10].

## Methods

### Study aim and design

The objectives of this study were to compare the **cobas** CT/NG assay on the **cobas®** 6800 system, with the Aptima Combo 2 assay on the Panther system, evaluating the high-throughput workflow and assay correlation in female urine specimens. This retrospective study was conducted at a single site (Labor Stein), a laboratory within the Limbach Group, located in Mönchengladbach, Germany, and performed in accordance with relevant local legislation. Remnant de-identified first-catch female urine specimens were collected between March and October 2017. Eligible samples were from symptomatic and asymptomatic individuals (> 18 years of age) presenting for CT/NG testing according to standard practice in North Rhine Westphalia, Germany.

The workflow and analytical performance of two commercially available assays were evaluated on their respective platforms: the **cobas** CT/NG on the **cobas** 6800 system with pre-analytics (**cobas p** 480) for de-capping and re-capping of specimens (Roche Molecular Systems Inc., Pleasanton, California, USA) and the Aptima Combo 2 on the Hologic Panther system (Hologic Inc., Marlborough, Massachusetts, USA). A pre-analytical de-capping, re-capping device was not required for the Panther system, as samples could be directly loaded onto the machine.

Both systems were available at the testing site. All assays were performed in accordance with the manufacturers’ instructions for use [9, 10]. Sample size calculations for the workflow portion of the study were based on the daily throughput of the instruments. Previously tested specimens were used to assess the performance of the **cobas** CT/NG and Aptima Combo 2 assays. Given that the historical prevalence rate is low at the study site, it was understood that convenience sampling may not be representative of the local test population. Capturing, monitoring, and analyzing of the workflow-related study data were executed by an independent third-party vendor with experience in workflow studies. Operators performing the testing represented the average operator trained to test according to standard procedures and manufacturers’ recommendations.

### Clinical specimens

Remnant urine specimens were divided into two aliquots and transferred into the **cobas®** PCR Urine Sample Kit or the Aptima Urine Specimen Collection Kit. Samples were stored at either 4 °C (for testing with **cobas** CT/NG) or − 20 °C (for testing with Aptima Combo 2) for a maximum of 8 months.

### Workflow (operation and system metrics) and assay agreement

The first 376 specimens collected were used for the workflow evaluation, simulating high-volume throughput in a single day. Testing of the paired **cobas** and Aptima urine specimens occurred within 1 week of each other. On the first day, specimens were assessed on the **cobas** 6800 system using the **cobas** CT/NG assay. On the second day, paired specimens were tested on the Panther system using the Aptima Combo 2 assay. Using standardized data capture forms and detailed interviews with laboratory personnel, the following operation and system metrics were collected: system capabilities (cycle times, throughput, capacity); labor and resourcing requirements (hands-on time [HoT], sample preparation); and total time required to perform all activities for sample testing.

Agreement between the two tests was determined using clinical specimens from both the workflow and additional assay correlation runs (see Additional file 1). Defined positivity cut-offs were pre-specified as per manufacturers’ recommendations. Samples that could not be confidently called positive or negative by either system were excluded from analysis. Results from the routine original Aptima Combo 2 testing were not available to the operators at the time of workflow and assay agreement testing.

### Discrepant resolution

Analysis of discrepant specimens, defined as specimens with discordant results between the two tests, included evaluation of the result signal (relative light unit [RLU] for Aptima Combo 2 or cycle threshold [Ct] for **cobas** CT/NG) and reference to the Aptima Combo 2 result generated during routine testing.

### Statistical analysis

No statistical analysis of the workflow data was performed. For performance assessment, paired results from the **cobas** CT/NG and Aptima Combo 2 assays, for both CT and NG, were compared and two-sided 95% confidence intervals (CIs) calculated to provide estimates of positive percent agreement (PPA), negative percent agreement (NPA), and overall percent agreement (OPA) between the tests. McNemar’s test was used for significance testing. All statistical analyses were performed using SAS software (SAS Institute, Cary, North Carolina, USA).

## Results

In total, 606 remnant de-identified female urine specimens were evaluated (*n* = 376 for workflow studies, *n* = 606 for method correlation; see Additional file 1). Of these, 50 were excluded from the method correlation analysis because of suspected contamination at the time enrolled specimens were aliquoted for the study (supported by referencing the original Aptima Combo 2 result). Contamination was suspected as high number of initially singularly CT positive samples (tested with the Panther system), collected during the same period, then tested positive for both CT and NG (as tested by both methods). In total 556 specimens were evaluable for method correlation analysis.

### Workflow operation and system metrics

Table 1 summarizes the HoT, number of interventions, time to first result, number of specimens at first result, and total turnaround time. The **cobas** 6800 system required eight manual interactions, comprising the following: 00:08:48 (hr:min:sec) pre-analytics start-up, 00:18:50 molecular system start-up and 00:43:59 ongoing sample processing time (total HoT = 01:11:37). Two manual interactions had the risk for cross-contamination. Once when the de-capped tubes were loaded onto the **cobas** 6800 system, then once when the de-capped specimens were removed post analysis and placed on the **cobas p** 480 for re-capping. Cross-contamination at this stage could be problematic if there is a need to re-test samples at a later time, for example to check for additional pathogens.

**Table 1:** Workflow comparison simulating high-volume CT/NG testing (*n* = 376)^*^

System	Hands-on time (hr:min:sec)	Number of interventions	Time to first result (hr:min:sec)	Number of specimens at first result^†^	Total turnaround time (hr:min:sec)
**cobas** 6800 system	01:11:37	8	02:53:00	94 specimens, 2 controls	07:45:26
Panther system	01:09:53	6	03:28:29	3 specimens, 2 controls	10:47:30

^*^Utilizing the **cobas** CT/NG for use on the **cobas** 6800 system and Aptima Combo 2 on the Panther system

^†^ For the Panther system, continuous loading results in an additional 5 results every 5 min

For the same workload, the Panther system required six manual interactions, comprising the following: 00:30:43 system start-up and 00:39:10 ongoing sample processing time (total HoT = 01:09:53). Four manual interactions had the risk for cross-contamination. Two of these interactions occurred during reagent preparation and two during the removal of specimens, reagents, and waste.

### Assay performance correlation

Paired urine specimens were tested on both the **cobas** CT/NG and the Aptima Combo 2 assays. Overall agreement between the two assays was > 99% for both CT and NG (Tables 2 and 3). McNemar’s test revealed no statistically significant difference between the assays (*p* = 1).

**Table 2:** Assay performance simulating high-volume CT/NG testing (*n* = 555^*^), results for *Chlamydia trachomatis*^†^

		Aptima Combo 2		
		CT positive	CT negative	Total
cobas CT/NG	CT positive	31	1	32
	CT negative	0	523	523
	Total	31	524	555
PPA (95% CI), NPA (95% CI)		100 (88.8, 100)	99.8 (98.9, 100)	
OPA (95% CI)		99.8 (99, 100)		
*p*-value^‡^		1		

^*^One CT specimen was not included in the CT method correlation because of an equivocal result generated by the Aptima Combo 2 assay (cobas CT/NG: “CT Positive” and Aptima Combo 2: “CT UNBEST”)

^†^Comparison of **cobas** CT/NG for use on the **cobas** 6800 system and Aptima Combo 2 on the Panther system

^**‡**^Calculated using McNemar’s test

Abbreviations: *CI* confidence interval, *CT Chlamydia trachomatis*, *NPA* negative percent agreement, *OPA* overall percent agreement, *PPA* positive percent agreement

**Table 3:** Assay performance simulating high-volume CT/NG testing (*n* = 556), results for Neisseria gonorrhoeae^*^

		Aptima Combo 2		
		NG positive	NG negative	Total
cobas CT/NG	NG positive	4	1	5
	NG negative	0	551	551
	Total	4	552	556
PPA (95% CI), NPA (95% CI)		100 (39.8, 100)	99.8 (99, 100)	
OPA (95% CI)		99.8 (99, 100)		
p-value^†^		1		

^*^Comparison of **cobas** CT/NG for use on the **cobas** 6800 system and Aptima Combo 2 on the Panther system

^†^Calculated using McNemar’s test

Abbreviations: *CI* confidence interval, *NG Neisseria gonorrhoeae*, *NPA* negative percent agreement, *OPA* overall percent agreement, *PPA* positive percent agreement

One specimen was positive by the **cobas** CT/NG assay and negative by the Aptima Combo 2 for CT (**cobas** CT/NG Ct = 39.69; Aptima Combo 2 RLU = 12); reference to the original Aptima Combo 2 result confirmed CT positivity (RLU = 479), in agreement with the **cobas** CT/NG result.

There was also one specimen that was **cobas** CT/NG positive and Aptima Combo 2 negative for NG (**cobas** CT/NG Ct = 35.77; Aptima Combo 2 RLU = 616): reference to the original Aptima Combo 2 result confirmed NG negativity (RLU = 983), in accordance with the Aptima Combo 2 result. Of note, this specimen was also positive for CT by both the **cobas** CT/NG and Aptima Combo 2 assays (**cobas** CT/NG Ct = 33.70; Aptima Combo 2 RLU = 616). No additional discrepant testing was performed.

## Discussion

National screening programs are an important aspect of STI prevention and support the need for high-volume testing systems [2, 5–8]. The **cobas** 6800 system and the Panther system are commonly used high-volume automated molecular platforms for routine CT/NG testing. Workflow analysis showed that the **cobas** 6800 system and the Panther system had similar HoT and number of manual interventions. However, the workflow analysis found the number of manual interventions with risk of a cross-contamination event was fewer with the **cobas** 6800 system compared with the Panther system (2 versus 4 events), mainly due to the ready-to-load reagents that did not require manual reconstitution and mixing. While the **cobas p** 480 is needed for the sample de-capping, the ready-to-load reagents of the **cobas** 6800 system can go directly on the system without pre-analytical preparation in a separate clean area, thus providing workflow efficiencies. The pierceable caps utilized on the Panther system are convenient for direct loading of specimens; however, after assaying, the tubes should be sealed with a barrier for storage [10]. The de-capping and re-capping of specimens with the **cobas p** 480 mitigates the risk of potential source contamination from direct contact with the foil caps during specimen handling, both pre-analytically in the clinic or in transit, as well as pre-analytically in the laboratory [10].

In other aspects, the **cobas** 6800 system had specific throughput advantages in comparison with the Panther system. The time to first result was sooner for a larger number of samples and, overall, the results were delivered 3 h (28%) faster on the **cobas** 6800 system.

Method correlation between the **cobas** CT/NG and the Aptima Combo 2 assays demonstrated excellent agreement, suggesting that both assays and platforms can be confidently used to screen for infections in a high-throughput setting. Although two specimens generated discrepant results, this was likely due to low titers of the microorganisms in the specimens.

Whilst this study has some limitations, including the use of convenience sampling, it is a useful tool to evaluate workflow differences and will aid decision making in clinical microbiology laboratories seeking to implement high-volume STI screening protocols. Additionally, the **cobas** 6800 system has more onboard test accessibility than the Panther system (12 polymerase chain reaction **cobas** assays versus four transcription-mediated amplification Hologic assays, respectively) [11]. Of note, the Panther Fusion system allows for additional menu but does not expand access to the core assays [11]. Advances in high-throughput molecular systems have provided solutions for laboratory consolidation and increasing laboratory services.

## Conclusion

The **cobas** 6800 system and the Panther system both provide improved automation with comparable performance for CT/NG testing. The **cobas** 6800 system, when utilized with the **cobas p** 480 pre-analytic system, would allow a growing laboratory service to deliver more results in a single shift. When deciding on automated molecular platforms laboratories will need to balance the needs of their workflow, resources, and service demands.

## Supplementary information

**Additional file 1.** “Illustration of testing and specimens evaluated for (A) workflow and (B) performance”, flow chart. (PPTX 43 kb)

## Data Availability

The datasets used and/or analysed during the current study are available from the corresponding author on reasonable request.

## Abbreviations

CI: Confidence intervals
CT: *Chlamydia trachomatis*
Ct: Cycle threshold
HIV: Human immunodeficiency virus
HoT: Hands-on time
NG: *Neisseria gonorrhoeae*
NPA: Negative percent agreement
OPA: Overall percent agreement
PPA: Positive percent agreement
RLU: Relative light unit
STIs: Sexually transmitted infections
