# Supplementary material for: High-volume workflow and performance comparisons for Chlamydia trachomatis and Neisseria gonorrhoeae testing using automated molecular platforms
Source: BMC Infect Dis. 2019 Sep 11;19:797. doi: 10.1186/s12879-019-4442-0 (PMC6737607; doi:10.1186/s12879-019-4442-0)
Supplement: Supplementary file 1 — Additional file 1. “Illustration of testing and specimens evaluated for (A) workflow and (B) performance”, flow chart. (PPTX 43 kb) [file 12879_2019_4442_MOESM1_ESM.pptx]

## Slide 1
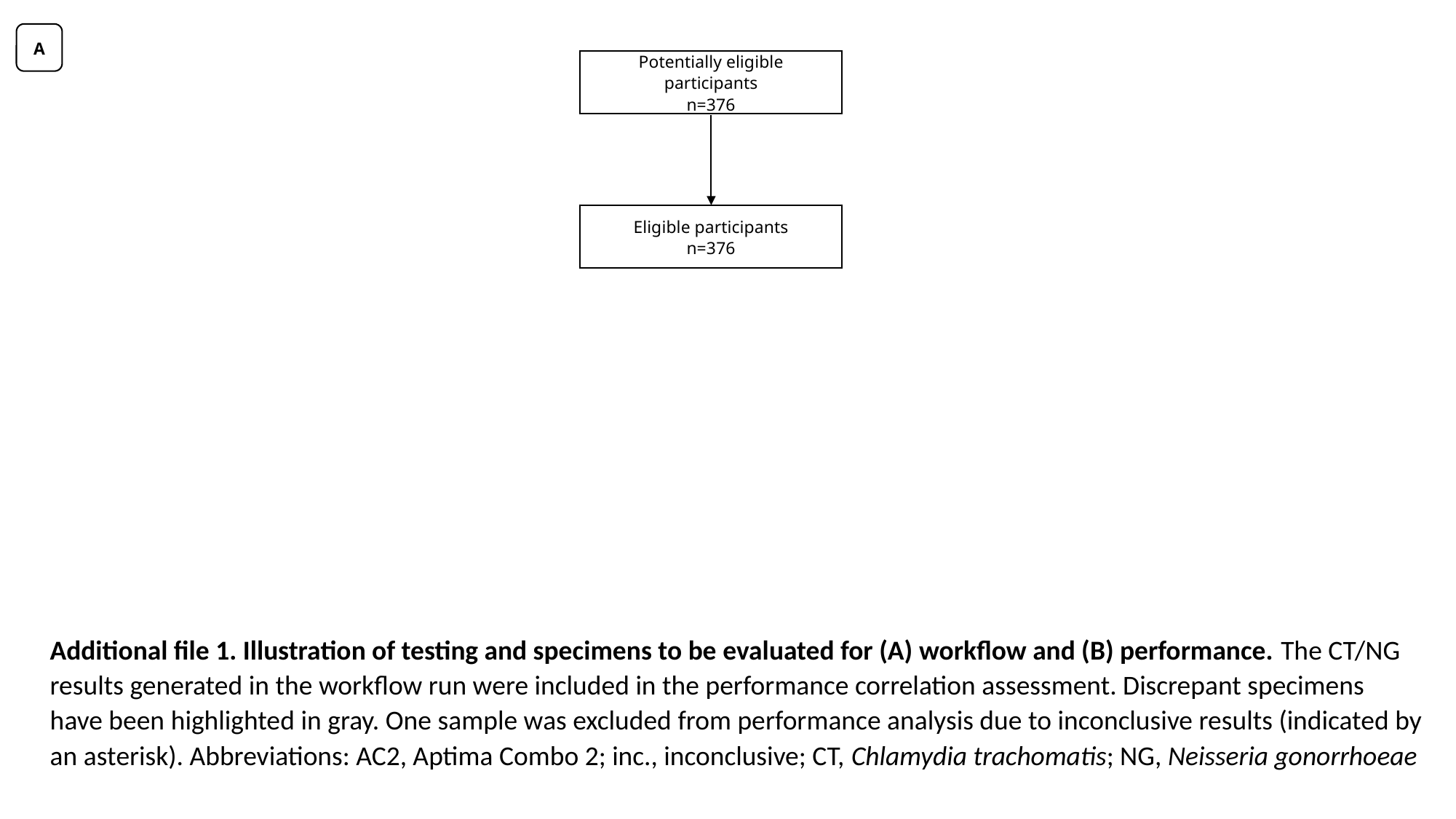

A
Potentially eligible participants
n=376
Eligible participants
n=376
Additional file 1. Illustration of testing and specimens to be evaluated for (A) workflow and (B) performance. The CT/NG results generated in the workflow run were included in the performance correlation assessment. Discrepant specimens have been highlighted in gray. One sample was excluded from performance analysis due to inconclusive results (indicated by an asterisk). Abbreviations: AC2, Aptima Combo 2; inc., inconclusive; CT, Chlamydia trachomatis; NG, Neisseria gonorrhoeae

## Slide 2
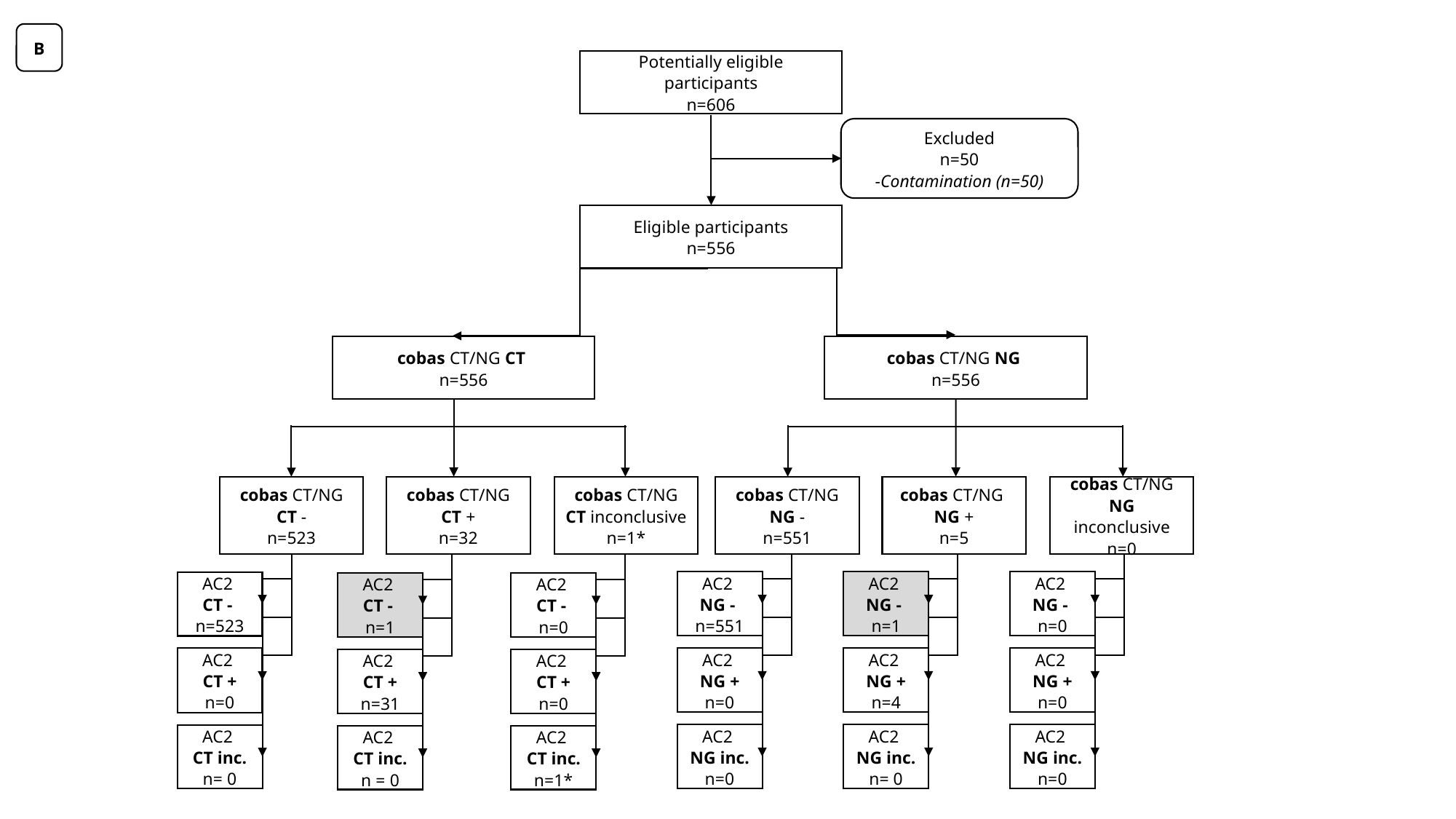

B
Potentially eligible participants
n=606
Excluded
n=50
-Contamination (n=50)
Eligible participants
n=556
cobas CT/NG CT
n=556
cobas CT/NG NG
n=556
cobas CT/NG CT -
n=523
cobas CT/NG CT +
n=32
cobas CT/NG CT inconclusive
n=1*
AC2
CT -
n=523
AC2
CT +
n=0
AC2
CT inc.
n= 0
AC2
CT -
n=1
AC2
CT +
n=31
AC2
CT inc.
n = 0
AC2
CT -
n=0
AC2
CT +
n=0
AC2
CT inc.
n=1*
cobas CT/NG NG -
n=551
cobas CT/NG
NG +
n=5
cobas CT/NG NG inconclusive
n=0
AC2
NG -
n=551
AC2
NG +
n=0
AC2
NG inc.
n=0
AC2
NG -
n=1
AC2
NG +
n=4
AC2
NG inc.
n= 0
AC2
NG -
n=0
AC2
NG +
n=0
AC2
NG inc.
n=0
